# Validation of Self‐Reported Gait Speed and Grip Strength Measures With Objective Measures in Older Adults With Mild Frailty in England

**DOI:** 10.1155/jare/1775375

**Published:** 2026-10-13

**Authors:** Zhengyi Chen, Tasmin Rookes, Kate Walters, Rachael Frost, Sarah Kalwarowsky, Louise Marston

**Affiliations:** ^1^ Research Department of Primary Care and Population Health University College London London UK ucl.ac.uk; ^2^ Department of Public and Allied Health Liverpool John Moores University Liverpool UK ljmu.ac.uk

**Keywords:** activities of daily living, ageing, frailty, trial, validation

## Abstract

**Background:**

Clinical research often relies on in‐person assessments, which can limit participation among frailer older adults who have difficulty attending clinical settings. Developing valid self‐reported alternatives could improve inclusivity. We aimed to validate self‐reported gait speed and grip strength against objective measures in adults aged ≥ 65 with mild frailty participating in the HomeHealth trial.

**Methods:**

Participants with paired subjective and objective gait speed and/or grip strength measures were included. Internal consistency was assessed using Cronbach’s alpha for gait speed (4 items) and grip strength (2 items), stratified by sex. Criterion validity was evaluated using sensitivity and specificity of self‐reported measures to identify frailty (Fried phenotype thresholds). Construct validity was examined through regression analyses with instrumental activities of daily living and quality of life outcomes.

**Results:**

A total of 254 participants were included (161 females, 93 males). Sensitivity and specificity for detecting slow gait speed were 65.5% and 65.8% in men and 79.5% and 59.0% in women. For weak grip strength, sensitivity and specificity were 23.0% and 66.7% in men and 49.3% and 93.3% in women. Internal consistency was low (*α* = 0.314–0.574 across measures). Construct validity was moderate, with expected associations observed for functional and quality of life outcomes.

**Conclusions:**

Self‐reported gait speed and grip strength measures demonstrated limited validity compared with objective assessments in older adults with mild frailty and should be used cautiously as substitutes for measuring weakness and slowness in this population.

**Trial Registration:** ISRCTN Registry: ISRCTN54268283

## 1. Introduction

It is important that all potential participants can participate in clinical research studies. For some, such as older participants unable to travel far from their home or those who are clinically vulnerable, this can be problematic, as they may struggle to attend research sites for assessments. When objective clinical measures are used, which require in‐person appointments, this can be a barrier to participation. Whilst home visits can be an alternative, these are often resource‐intensive and may present further difficulties in rural and remote areas. Finding a way to adapt our research measures to promote inclusion and reduce sampling bias is essential. Unpublished data from a trial in mildly frail older people (HomeHealth) showed that telephone and video research appointments were acceptable to this population.

Frailty is increasing with 12% of those aged over 60 worldwide experiencing physical frailty; 15% in females and 11% in males and the prevalence increases with age [1]. Those who are frail are at greater risk of hospitalisations and move to long‐term care and have greater resource use [2, 3]. In recent years, older people with frailty are the target population in many studies aiming to promote independence and prevent and/or reduce frailty. However, those who are frailer are less likely to be able to complete measures which require face‐to‐face appointments, especially away from the home. In clinical practice, it may not be possible or practical to carry out objective measures of physical frailty as they can be time consuming or require specialist equipment with a lot of space, so quicker and easier alternatives would be advantageous.

Assessments of older adults tend to focus on activities of daily living, rather than gait speed and grip strength domains, which are components of the frailty phenotype. The most used frailty measure, the Fried Frailty Phenotype [4], considers frailty in five domains: unintentional weight loss, exhaustion, weakness (grip strength), slow walking speed, and low physical activity. Each domain is measured separately and is dichotomised into a score of 1 or 0 point based on prespecified cutoff values. An overall score of 3+ indicates frailty on the phenotype, a score of 1–2 indicates prefrailty, and a score of 0 indicates a person is robust. In the original Fried Frailty Phenotype, weakness and walking speed were measured objectively by a researcher and the other domains are assessed through questionnaires. Many other studies have used different subjective questions to measure weakness and walking speed, including incorporating questions about instrumental activities of daily living [5], though a consensus on which were the best questions has not been reached. The objective measure of walking speed varies too, although is often 3–6 metres in research studies. The distance is not always able to be standardised, meaning that over longer distances participants may become tired and over shorter distances people may not have got up to speed [5].

Two studies investigated and validated the optimal questions to ascertain subjective grip strength (weakness) and walking speed in Belgium and the Netherlands [6, 7] in place of the objective measures in the Fried Frailty Phenotype. Two questions were established for grip strength and four for walking speed. A high concordance between the objective and subjective measures of grip strength and walking speed with high specificities was found for those older people with and without frailty [6]. They found a sensitivity of 69% and specificity of 86% for walking speed and sensitivity of 73% and specificity of 71% for grip strength [6].

The aims of the current study are to validate the self‐reported gait speed questions against objective gait speed and to validate self‐report grip strength questions against objective grip strength in mildly frail older people in England.

## 2. Methods

This paper examines the criterion and construct validity and the internal consistency of the self‐reported measures and has been written in accordance with the STARD guidelines [8].

### 2.1. Study Population

This study drew on a sample of participants from the HomeHealth trial [9], a randomised controlled trial of a 6‐month home‐based intervention to maintain independence in older people with mild frailty versus treatment as usual. Data were collected at baseline, 6 months and 12 months after randomisation by a researcher either face‐to‐face in participants’ homes, by telephone or by videoconferencing. Objective measures of gait speed and grip strength could only be collected in home‐based appointments, and therefore, only data from these appointments are included in this analysis. The trial found no difference between randomised groups in grip strength or gait speed at any time point [10], so no distinction between randomised groups was made. To be included in this analysis, participants needed to have a paired objective and subjective measure of grip strength or gait speed at any time point. Only one time point per participant was used. Across the three time points, 12‐month data were preferred because this was the HomeHealth primary outcome time point. If participants did not have 12‐month data, then data at 6‐month or baseline, respectively, were used.

### 2.2. Study Procedures

#### 2.2.1. Gait Speed

Gait speed was measured objectively with a timed walk using a stopwatch between 2 and 5 metres depending on the length of the available path in the participant’s home [11]. Participants were given 0.5–1 m either side for speeding up and slowing down, which was not timed. Participants had up to two trials of this assessment. They could use walking aids if necessary and were instructed to walk at their usual pace. Distance and time were used to calculate speed in metres/second. For analysis, we took the mean of the two gait speed measurements or the one measurement if only one was conducted. Gait speed 0.73 m/s or slower for men and 0.65 m/s or slower for women was considered the cutoff for slowness based on the U.K. norms from the English Longitudinal Study of Ageing [12]. These speeds are the mean of the two height categories by sex; actual height category cutoffs could not be used as HomeHealth did not collect data on participant height.

The subjective measure of gait speed used the four established and validated questions in older Flemish and Dutch populations [6, 7]. The questions were interviewer‐administered. Points are given in brackets, with higher scores indicating greater difficulty. A score of 0 represents no frailty and between 3 and 5 indicates meeting the cutoff for a frailty point in the Fried phenotype in terms of gait speed.

1. When the doorbell rings, do you usually get there in time to open the door? (no = 1, yes = 0).
2. Do you walk more slowly than you’d like? (yes = 2, no = 0).
3. Do you have enough time to cross the street on foot when the traffic light turns green? (no = 1, yes = 0).
4. Do you encounter problems in daily life due to poor balance? (yes = 1, no = 0).

#### 2.2.2. Grip Strength

Grip strength was measured objectively as the gold standard using a dynamometer, following the standardised protocol outlined by Roberts et al. [13]. Participants were seated with their forearm resting on a chair arm or table. Each participant was given up to three attempts on each hand. For analysis, we used the highest value of the stronger hand. Grip strength ≤ 36.5 kg for men and ≤ 22.0 kg for women was considered the cutoff for weakness, based on cutoffs from the U.K. norms from the English Longitudinal Study of Ageing [14]. These cutoffs are the mean of the two cutoffs for mid BMI categories as height and weight data were not collected in HomeHealth [12].

Grip strength was measured subjectively using two questions which were established and validated in older Flemish and Dutch populations [6, 7]. Points for positive responses are in brackets, with higher scores indicating greater weakness and a score of 1, 2 or 3 indicates a point for frailty in the Fried phenotype in terms of grip strength.

1. Do you have trouble watering plants with a spray bottle? (yes = 2, no = 0).
2. Do you feel like you have less hand strength than other people your age? (yes = 1, no = 0).

### 2.3. Statistical Methods

Baseline demographics and variables measured for those eligible for this study were summarised using descriptive statistics. Continuous variables were described using median (IQR). Categorical variables were described using frequencies (%). Analyses were carried out separately for males and females because of differing cutoffs for frailty point on objective measures and to determine if there was a difference in the suitability of the cutoffs by sex.

Cronbach’s alpha [15] was calculated for the gait speed and grip strength questions separately. Acceptable values range from 0.75 to 0.95 [16]. For criterion validity, we examined sensitivity, specificity, positive and negative predictive values and Cohen’s kappa, all with 95% CI, between the gold standard (objective) cutoffs and the questionnaire cutoffs for frailty [17–19].

To further assess construct validity of subjective gait speed and grip strength, we carried out linear regression analyses with Nottingham Extended Activities of Daily Living (NEADL) [20, 21] and EQ‐5D‐5L utilities to assess health quality of life [22] at the same time point as the grip strength and gait speed were assessed as outcomes. For these outcomes, higher scores indicate greater independence in instrumental activities of daily living and better quality of life, respectively. Subjective gait speed and grip strength scores were the explanatory variables (separately), adjusting for site. Analyses were carried out using Stata Version 18 [23].

## 3. Results

Data were available for 254 participants to be included in this analysis (Figure 1). Most participants had paired data at the 12‐month follow‐up (183/254) (Table 1). There were 161 females and 93 males. A total of 173 (68%) participants for gait speed and 110 (43%) participants for grip strength met the frailty cutoff with both objective and subjective scores. Overall, the sample was representative of deprivation levels, education levels, home ownership and sexuality. Our sample was slightly older, had more females and less people from ethnic minorities [24]. Table 2 shows the regression outcomes, objective and subjective grip strength and gait speed by gender.

**FIGURE 1:**
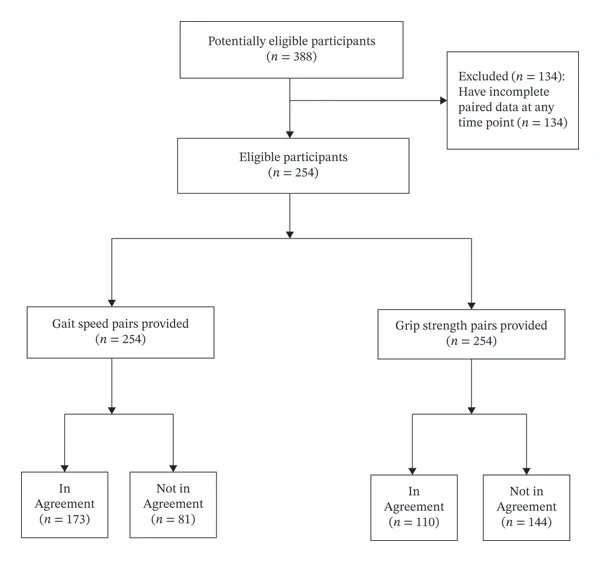
STARD flow diagram.

**TABLE 1:** Descriptive statistics.

Characteristic	Median (IQR) or N (%)
Age (years)	83 (78, 87)
Gender	
Male	93 (36.7%)
Female	161 (63.3%)
Site	
UCL/Camden	71 (28.0%)
UH/Herts	93 (36.6%)
BTHFT/Yorks	90 (35.4%)
Ethnicity	
White	241 (94.9%)
Asian	7 (2.8%)
Other	6 (2.3%)
Birthplace	
UK	221 (87.0%)
In another country	33 (13.0%)
Sexuality	
Heterosexual/straight	248 (97.6%)
LGB (+other)	3 (1.2%)
Missing	3 (1.2%)
Living arrangements	
I live alone	144 (56.7%)
I live with my spouse/partner	89 (35.0%)
I live with my children	8 (3.2%)
I live with a friend/other	13 (5.1%)
Marital status	
Single	16 (6.3%)
Cohabiting with partner/married/in a civil partnership	98 (38.6%)
Separated/divorced	31 (12.2%)
Widowed	107 (42.1%)
Other	2 (0.8%)
Housing	
Owner‐occupied	179 (70.5%)
Council rented	22 (8.7%)
Housing association rented/social housing rented	25 (9.9%)
Private rented	9 (3.5%)
Sheltered housing	10 (3.9%)
Other	9 (3.5%)
Education level	
No formal qualifications	91 (35.8%)
General certificate of education/O level or equivalent	51 (20.1%)
Certificate of secondary education/A level or equivalent	20 (7.9%)
Higher national diploma or equivalent	28 (11.0%)
Degree or higher degree	64 (25.2%)
Current employment status	
Employed	5 (2.0%)
Retired	247 (97.2%)
Other	2 (0.8%)
State pension	
Yes	253 (99.6%)
No	1 (0.4%)
Employer pension	
Yes	114 (44.9%)
No	140 (55.1%)
Private pension	
Yes	106 (41.7%)
No	148 (58.3%)
Smoking status	
Yes, I currently smoke or use tobacco	7 (2.8%)
No, I have never smoked or used tobacco	105 (41.3%)
No, but I have smoked or used tobacco in the past	142 (55.9%)
Time point data used	
12 month	183 (72.1%)
6 month	32 (12.6%)
Baseline	39 (15.3%)

**TABLE 2:** Comparison of baseline descriptive statistics between males and females.

Descriptive statistics mean (SD)	Males (*N* = 93)	Females (*N* = 161)
NEADL score	43.59 (12.91)	45.89 (10.26)
EQ‐5D‐5L utility units	0.73 (0.18)	0.71 (0.20)
Subjective gait speed score	2.57 (1.54)	2.62 (1.37)
Subjective grip strength score	0.39 (0.79)	0.94 (1.15)
Objective gait speed (m/s)	0.65 (0.28)	0.71 (0.47)
Objective grip strength (kg)	24.22 (8.10)	13.89 (6.58)

*Note:* For objective measures, lower scores indicate higher frailty. For subjective measures, higher scores indicate more severe frailty.

For males, the gait speed subjective questionnaire was more internally consistent, compared to grip strength (Cronbach’s alpha 0.574 and 0.314, respectively). Females alpha scores for gait speed were 0.440 and 0.359 for grip strength. This indicates low to moderate internal consistency for the subjective measures (Table 3).

**TABLE 3:** Cronbach’s alpha for internal consistency of subjective questions by gender.

Validated variable name	Cronbach’s alpha
Male	
Gait speed (4 questions)	0.574
Grip strength (2 questions)	0.314
Female	
Gait speed (4 questions)	0.440
Grip strength (2 questions)	0.359

Using the cutoffs, we defined weakness/slowness determined by objective measures as true positive and weakness/slowness determined by subjective scores as test positive. For male gait speed (Table 4), 65.6% showed measures agreement, while the sensitivity was 65.5% and specificity was 65.8%. For male grip strength, 25.8% showed measures agreement, sensitivity was 23.0% and specificity was 66.7%. For female gait speed, measures agreement was 69.6%, the sensitivity was 79.5% and specificity was 59.0%. For female grip strength, measures agreement was 53.4%, sensitivity was 49.3% and specificity was 93.3%. Criterion validity was, therefore, limited for both measures and varied by gender.

**TABLE 4:** Criterion validation statistics by gender.

Validation statistics for males		
Variable names	Value	95% CI
Gait speed		
Sensitivity	65.5%	(51.4%, 77.8%)
Specificity	65.8%	(48.6%, 80.4%)
Positive predictive values	73.5%	(58.9%, 85.1%)
Negative predictive values	56.8%	(41.0%, 71.7%)
Cohen’s Kappa	0.305	(0.104, 0.506)
Measures in agreement	61 (65.6%)	N/A
Grip strength		
Sensitivity	23.0%	(14.6%, 33.2%)
Specificity	66.7%	(22.3%, 95.7%)
Positive predictive values	90.9%	(70.8%, 98.9%)
Negative predictive values	5.6%	(1.6%, 13.8%)
Cohen’s Kappa	−0.017	(−0.025, 0.048)
Measures in agreement	24 (25.8%)	N/A

**Validation statistics for females**		

**Variable names**	**Value**	**95% CI**

Gait speed		
Sensitivity	79.5%	(69.2%, 87.6%)
Specificity	59.0%	(47.3%, 70.0%)
Positive predictive values	67.3%	(57.1%, 76.5%)
Negative predictive values	73.0%	(60.3%, 83.4%)
Cohen’s Kappa	0.387	(0.235, 0.539)
Measures in agreement	112 (69.6%)	N/A
Grip strength		
Sensitivity	49.3%	(41.0%, 57.7%)
Specificity	93.3%	(68.1%, 99.8%)
Positive predictive values	98.6%	(92.6%, 100%)
Negative predictive values	15.9%	(9.0%, 25.2%)
Cohen’s Kappa	0.134	(−0.0075, 0.041)
Measures in agreement	86 (53.4%)	N/A

For males, Cohen’s kappa was 0.305 for gait speed and −0.017 for grip strength, indicating low agreement. Results for females were slightly better, but agreement was still low: 0.387 for gait speed and 0.134 for grip strength (Table 4).

Table 5 shows the coefficients for continuous subjective scores. For males, a 1‐point increase in subjective gait speed score was associated with a reduced NEADL score and EQ‐5D‐5L utility by 3.46 (−4.91, −2.02) and 0.061 (−0.082, −0.041), respectively. A 1‐point increase in grip strength score was associated with a lower NEADL score and EQ‐5D‐5L utility by 2.99 (−6.01, 0.02) and 0.063 (−0.107, −0.018), respectively. For females, a 1‐point increase in gait speed score was associated with a lower NEADL score and EQ‐5D‐5L utility by 3.40 (−4.36, −2.44) and 0.064 (−0.085, −0.043), respectively. A 1‐point increase in grip strength score was associated with a lower NEADL score and EQ‐5D‐5L utility by 1.83 (−3.08, −0.59) and 0.049 (−0.076, −0.023), respectively. The questions therefore showed reasonable construct validity.

**TABLE 5:** Linear regression analyses to assess construct validity by gender.

Coefficient estimates with 95% CI for males (*N* = 93)		
Outcome	Unadjusted	Adjusted for sites
NEADL (N = 92)		
Subjective gait Speed	−3.80 (−5.38, −2.21)	−3.46 (−4.91, −2.02)
Subjective grip Strength	−3.30 (−6.62, 0.02)	−2.99 (−6.01, 0.02)
Objective gait speed	26.55 (18.57, 34.52)	24.93 (16.99, 32.87)
Objective grip strength	0.40 (0.08, 0.72)	0.26 (−0.04, 0.57)
EQ‐5D‐5L Utility (N = 93)		
Subjective gait speed	−0.062 (−0.082, −0.042)	−0.061 (−0.082, −0.041)
Subjective grip strength	−0.064 (−0.108, −0.020)	−0.063 (−0.107, −0.018)
Objective gait speed	0.349 (0.238, 0.459)	0.383 (0.264, 0.501)
Objective grip strength	0.001 (−0.004, 0.005)	0.000 (−0.005, 0.005)

**Coefficient estimates with 95% CI for females (*N* = 161)**		

NEADL (N = 159)		
Subjective gait speed	−3.85 (−4.88, −2.83)	−3.40 (−4.36, −2.44)
Subjective grip strength	−2.40 (−3.75, −1.05)	−1.83 (−3.08, −0.59)
Objective gait speed	9.06 (5.89, 12.23)	6.89 (3.79, 9.99)
Objective grip strength	0.53 (0.29, 0.76)	0.27 (0.02, 0.52)
EQ‐5D‐5L Utility (N = 161)		
Subjective gait speed	−0.068 (−0.088, −0.047)	−0.064 (−0.085, −0.043)
Subjective grip strength	−0.054 (−0.080, −0.028)	−0.049 (−0.076, −0.023)
Objective gait speed	0.138 (0.074, 0.203)	0.122 (0.054, 0.189)
Objective grip strength	0.010 (0.005, 0.015)	0.009 (0.004, 0.014)

*Note:* Where the outcome is NEADL, the coefficients are in NEADL score; where the outcome is EQ‐5D‐5L, the coefficients are in EQ‐5D‐5L utility units.

Abbreviations: EQ‐5D‐5L, EuroQol 5 Dimensions, 5 Levels; NEADL, Nottingham Extended Activities of Daily Living.

## 4. Discussion

Findings from 254 participants suggest that the self‐reported measures of weakness and slowness evaluated in this study show low internal consistency and are notagreeable with objective measures, in both male and female older adults living in England with mild frailty.However, the self‐reported measures showed reasonable construct validity. As scores on the subjective measures for grip strength and walking speed worsened, independence and quality of life scores decreased as expected. Self‐reported prevalences of slowness and weakness were more than 10% higher for females than males, which could represent higher prevalence of frailty in females or that females may be more likely to report their frailty as worse than it is and males may be more likely to self‐report that their frailty is less severe than it is.

### 4.1. Results in Context

Despite previous validation studies suggesting that subjective measures of slowness and weakness are valid and agree with objective measures in Dutch and Flemish older populations with a range of frailty levels [6, 7], when evaluated in participants living in England with mild frailty, we have not been able to replicate these findings. One potential explanation for this discrepancy is that the mean age of participants in the HomeHealth trial, and therefore this analysis, are older than the other studies which have validated the subjective measures(81 (SD 6.5) years in comparison to 73 (SD 8.0) and 74 (SD 7.0)) [6, 7]. Therefore, the subjective measures may have greater validity in younger populations, where weakness and slowness are likely to be less prevalent than in older populations. There may have also been cross‐cultural challenges, for example, one grip strength question discusses the use of spray water bottles, which is an activity more common in Dutch older adults than in England. Subjective feedback from researchers carrying out the assessments also indicated that participants found it difficult to judge whether they were less strong than other people.

Since the data were collected for this trial, new self‐report questions have been developed in the United States to measure slowness and weakness [25]. They highlight that the validity of self‐reported measures depends on how frail an individual is, which supports the argument that self‐report measures may have greater validity in younger, less frail participants than older, frailer participants. With the aim of self‐reported measures being to increase involvement of frailer people within clinical research, these findings suggest that currently available self‐reported questions to assess slowness and weakness should not be used as part of the Fried frailty phenotype in English older people. This is further exacerbated by the overreporting of frailty in females and underreporting in males [25]. It may, therefore, be of greater value to use an overall subjective self‐reported measure of frailty instead (e.g., Tilburg Frailty Indicator [26]).

### 4.2. Strengths and Limitations

This study utilised a large sample of participants, with data collected as part of a national randomised controlled trial which collected data at multiple time points over the course of a year. By offering the subjective measure of weakness and slowness, this both enabled the research to continue throughout the COVID‐19 pandemic and increased participation from people who were unable to attend assessment visits due to shielding or COVID‐19 restrictions, making our sample more representative.

It was not possible to evaluate test–retest reliability, as retest data would have needed to be collected within a month of the test data. The present data were collected at 6‐month intervals, giving time for grip strength and/or gait speed to change. Also, we did not have the participants’ height and weight values, which meant that the true reference cutoffs could not be calculated and assigned. Therefore, the cutoffs used to validate the subjective measures may be less accurate than desired, reducing the reliability of the results. Our sample was limited to people recruited as part of the HomeHealth trial, and grip strength and walking speed were not primary outcomes for the study. While 388 participants were recruited into the trial, data were only available for 254 participants due to the COVID‐19 pandemic and some assessments being conducted remotely. Demographics in this validation analysis were largely similar to the overall HomeHealth sample [10]. The HomeHealth sample of participants showed similar levels of deprivation, educational level and home ownership to the U.K. census data for older people in local areas, but fewer ethnic minorities and people born outside the UK [24], suggesting some generalisability of these results to the populations in these areas.

### 4.3. Future Research

We suggest that additional research be conducted to develop and validate self‐reported measures of weakness and slowness as part of the frailty phenotype for U.K. older adults who are frail. These new measures should also take into consideration the over‐ and under‐reporting of frailty in females and males, respectively. Once developed, it would be good to explore their implementation into clinical research to determine if they result in more diverse and inclusive study samples.

## 5. Conclusion

In a population of older adults living in England with mild frailty taking part in the HomeHealth trial, it was unclear how valid previously validated subjective measures of grip strength and gait speed were when compared to the objective measures collected. This could be due to the sample being older and perhaps frailer than those which have validated the measures previously or cross‐cultural differences in questions. Also, the potential subjective overreporting and underreporting of slowness and weakness in females and males, respectively, could have impacted the validity of the measures. Trials in England should be cautious when using these subjective scores in their current form as a way of measuring weakness and slowness as part of the frailty phenotype.

## Funding

This project was funded within the HomeHealth trial: NIHR Health Technology Assessment (NIHR128334).

## Disclosure

The views expressed are those of the authors and not necessarily those of the NIHR or the U.K. Department of Health and Social Care.

## Ethics Statement

The trial was reviewed and approved by the Health Research Authority Social Care Research Ethics Committee (20/IEC08/0013). All participants provided informed consent to take part in the trial and have their data collected and analysed. No additional consent was required for this secondary analysis of anonymised data.

## Conflicts of Interest

The authors declare no conflicts of interest.

## Data Availability

The data that support the findings of this study are available from the corresponding author upon reasonable request.
